# FishTrace: a genetic catalogue of European fishes

**DOI:** 10.1093/database/bax075

**Published:** 2017-10-31

**Authors:** Antonella Zanzi, Jann Th Martinsohn

**Affiliations:** 1 European Commission, Joint Research Centre (JRC), Directorate D–Sustainable Resources, via Enrico Fermi 2749, Ispra, VA 21027, Italy

## Abstract

FishTrace is a genetic catalogue for species identification associated to reference collections of taxonomically identified vouchers from more than 200 commercial marine fish species. The main purpose of the genetic catalogue is to enable reliable species identification for research purposes as well as in support of traceability schemes under the remit of food and feed laws. A major asset of FishTrace is that all genetic data are linked to biological collections of vouchers, that is the fish specimen that have been identified genetically have, at the same time, been identified by taxonomists and are stored and curated by natural history museums. This opens the potential for future applications related to fish species authenticity tests, also in a legal context, and associated biological research. The genetic catalogue, which contains molecular data together with detailed information on sampling and geographical origin, is publically accessible on the web site of the project.

**Database URL:**

https://fishtrace.jrc.ec.europa.eu

## Introduction

FishTrace was a four-year project (2002–06) funded by the European Union 5th Framework Programme in the area of ‘Quality of Life and Management of Living Resources’.

The rationale of FishTrace was the prevailing lack of readily available species identification tools, based on reliable references, that are required in fisheries management, biological and ecological research and also in traceability schemes underlying legal frameworks concerned with seafood safety [reviewed in Hofherr *et al.* (1)].

The main achievements of the project have been: the compilation of a comprehensive genetic catalogue encompassing all major commercially exploited European marine fish species, and the establishment of a collection of biological vouchers from taxonomically and genetically identified fish species for future use.

During the FishTrace project, 220 marine teleost fish species with commercial, ecological and zoological interest have been sampled from European sea areas.

Each specimen was morphologically identified, measured and photographed; afterwards biological samples (i.e. muscle tissues, DNA samples and otoliths) from the same specimens were obtained.

DNA samples were transferred to the FishTrace scientific groups for molecular genetic analysis. The results of the genetic analyses were used to build a catalogue containing molecular data together with detailed information on sampling and geographical origin.

The FishTrace genetic catalogue is based on two genes, one mitochondrial (cytochrome b) and one nuclear (rhodopsin), as molecular markers related to the morphological identified specimens. The choice of these two genes as satisfactory identification markers was tested in a previous project–PescaBase (2)–and in FishTrace has been confirmed its universal use for fish identification. Systematic resolution of cytochrome b and rhodopsin genes were settled by phylogenetic analyses including representative sequences from both genes directly retrieved from the FishTrace database and from NCBI GenBank^®^ (3), these last sequences were used as quality control in the analyses.

After taxonomic identification of the specimen, the biological samples obtained from it were permanently stored into biological collections hosted and curated by four European natural history museums. Collections stored in these museums comprise entire fish specimens, muscle tissues, otoliths and DNA samples that can be accessed for cross-referencing and as research resources for the identification of European commercial fish species.

These collections constitute a reference infrastructure with important applications in fish species authenticity and related biological research. Given the excellence and tradition of the host natural history museums, the long-term preservation and maintenance of these collections is guaranteed.

## Sampling and taxonomy

A total of 220 species belonging to 75 different families and 17 different higher teleostean orders were sampled [grade Teleostomi; class Actinopterygii; subclass Neopterygii; division Teleostei–based on a review by Nelson (4)]. These species are all marine forms with 9 marine-brackish species and 6 marine-brackish-freshwater species. The full list of the species considered is available on the project web site.

The distribution of species within teleost orders covered by the sampling tried to be representative of species availability and consumer’s demand in European markets; the rationale for the selection of sampled species also included their economic value and fisheries abundance.

Species identification was carried out by professional taxonomist applying morphological, chromatic and meristic approaches (the description of the adopted protocol is available on the project web site). For this purpose, the main UNESCO catalogues, the FAO guides and the major species identification catalogues have been used. Special attention has been paid to the traditional problems of differentiating adults of morphologically similar species and immature specimens of sister species within the same family.

**Figure 1.**
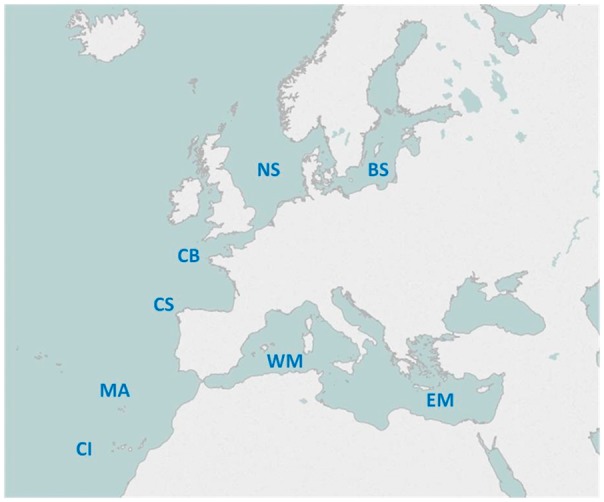
European sea areas covered by the project.

Eight European sea areas have been sampled to collect specimens from the targeted marine teleost species. From North to South, the European sea areas covered were (Figure 1): Skagerrak and the Baltic Sea (BS), the North Sea (NS), the English Channel and the Bay of Biscay (CB), the Cantabric Sea and the North-West Iberian Peninsula (CS), the Western Mediterranean Sea and Bay of Cadiz (WM), the Eastern Mediterranean Sea (EM), the Madeira Archipelago (MA) and the Canary Islands (CI).

**Figure 2.**
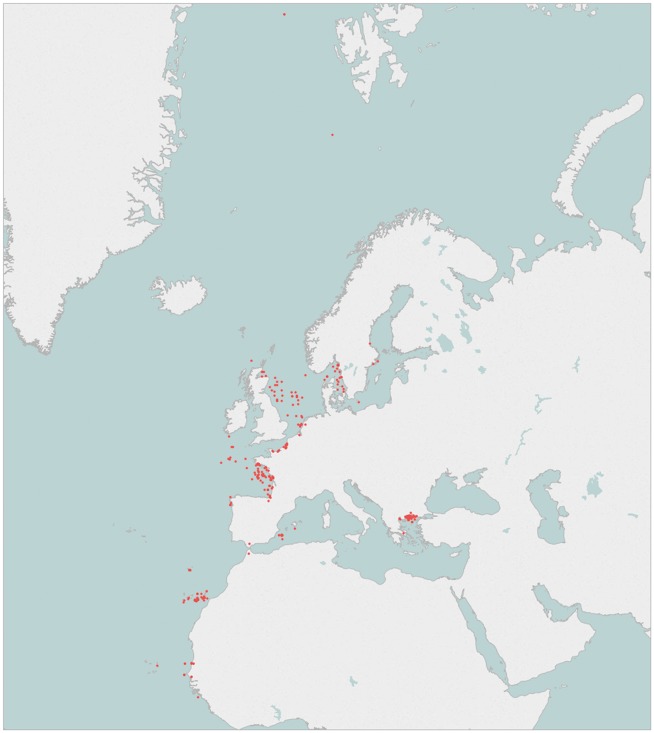
Geographical position of the sampling locations.

To elaborate the genetic catalogue, five specimens from each targeted species have been sampled at each geographical area covered. Many of the species overlap through the different geographical areas and therefore there are >100 species which are largely represented by more than ten specimens. The geographical position of the sampling locations is displayed in Figure 2.

Tissue samples were removed from each specimen sampled from the muscle behind the right pectoral fin base or from the muscle from inside the head in case of large fishes; they were conserved at –20 °C until their use for DNA extraction.

DNA samples were extracted from at least two of the five specimens collected from each sampling area. DNA from each specimen tissue sample was extracted following different protocols, depending on the laboratory in charge. Details on the DNA extraction method used for each sample was recorded and is available in the FishTrace database. 

## DNA sequences

The FishTrace catalogue provides information on the nucleotide sequences of the mitochondrial cytochrome b gene and the nuclear rhodopsin gene from the target species.

The analysis of the mitochondrial cytochrome b gene allows sample identification at species level; whereas, given the possible subtle genetic variation in populations at the mitochondrial level, the rhodopsin gene was used as a quality control procedure. The nuclear gene coding for rhodopsin shows minimal population variation in fish and is intronless in all teleost species. Thus this second marker allows for the validation of the analysis of the mitochondrial sequence and confers a degree of reliability required to quantify the level of divergence among species, while maintaining homogeneity in the same species.

When used in phylogenetic analyses, the combination of these two genes with different evolutionary rates serves to identify fish at the species level (5). Moreover, the given independent variation rate for each gene has allowed the identification of basal phylogenetic relationships and identify any rare case of paraphyly or hybridization between close species (6).

The genetic analyses were carried out at the following laboratories: French Research Institute for the Exploitation of the Sea (IFREMER), Hellenic National Agricultural Research Foundation (NAGREF), Swedish Museum of Natural History (NRM), Netherlands Institute for Fisheries Research (RIVO) and Complutense University of Madrid (UCM).

For the DNA barcoding of the targeted teleost specimens, the mitochondrial cytochrome b gene and the nuclear rhodopsin gene were Polymerase Chain Reaction (PCR) amplified. The length of the fragments amplified (1141 bp for cytochrome b and 460 bp for rhodopsin) guaranteed accuracy and efficiency in species identification by this molecular approach (7). The protocols underlying the genetic analyses are provided in detail in Sevilla *et al.* (5).

The validation process of the DNA sequences obtained implied: the comparison of DNA sequences obtained from two specimens of the same species at each geographical locations, and the verification of the position of each sequence on the accepted molecular phylogeny of the fish, through BLAST searching and calculating molecular phylogeny trees. In addition, all the sequences from a species were compared; the consistency of phylogenies and gene variability analysis of the set of sequences determined the validation of the species.

Sequences obtained and validated as DNA-barcodes during the FishTrace project were submitted to NCBI GenBank^®^.

## FishTrace collections of biological materials

The aim of the FishTrace collections of biological materials from taxonomically and genetically identified fish species is to be a reference infrastructure in Europe for studies related to fish species authenticity.

Four reference collection centres have been established: the National Museum of Natural History in Paris (MNHN); the Swedish Museum of Natural History in Stockholm (NRM); the Museum of Natural History in Tenerife (TFMC); the Institute of Marine Research–Municipal Museum in Funchal (IMAR–MMF). All four museums agreed to incorporate the FishTrace collections in their own ones and therefore keep them well preserved.

The reference collections include around 1770 entire fish specimens used to obtain molecular data (fixed in formalin and preserved in 70% ethanol or 50% isopropanol), muscular tissue samples from the same specimens (preserved in in 96% or 70% ethanol and kept refrigerated), replicate DNA samples from the same specimens (preserved frozen at –20 °C to –80 °C), and around 600 pairs of otoliths (dried).

The preservation of the biological materials in 70% ethanol guaranties its long-term conservation for cross-referring and molecular genetic analysis; this is confirmed by the reports that ancient DNA from museum specimen vouchers which has been stored for tens of years has been recovered from biological material fixed with formalin and preserved using ethanol (8, 9).

The otoliths collected, which are hard structures that are distinct for each species, can be used as additional reference for fish identification and data validation. Otoliths are the most widely used hard structures for fish identification, since they exhibit a high interspecific variability (10) and can be observed and studied from the larva stage of fishes. Furthermore, the otoliths collection represents another source of DNA since it can be easily extracted from this dry tissue.

The FishTrace consortium retained exclusive rights over the samples until June 30th, 2007. After that date, each museum’s policies started to apply to the collections.

Samples from the collections can be requested for the purpose of study. In order to request the loan of biological materials from the collections, a loan form (which is available on the project web site) has to be completed and directed to the curator of the collection where the chosen sample is stored.

Information on the different collections is available online on the web site of the project along with the identification codes for specimens, tissues, DNA samples and otoliths that can be used to request the samples from the curators of the collections.

## Database structure

Collected fish specimens were tagged with a unique FishTrace specimen code. The FishTrace code consists of a combination of the first three letters of the generic name, the first three letters of the specific name, two letters denoting geographical area, and two digits denoting the specimen number (e.g. the second specimen of *Mullus surmuletus* collected in the Western Mediterranean area has the following code: MulSur-WM-02). Species with identical first three letters in generic name and species epithet require an ad hoc code, which is constructed based on the first differing letter. In addition to this code, the biological materials stored in the collections are tagged with a collection identifier that is different from the unique FishTrace specimen code.

**Figure 3.**
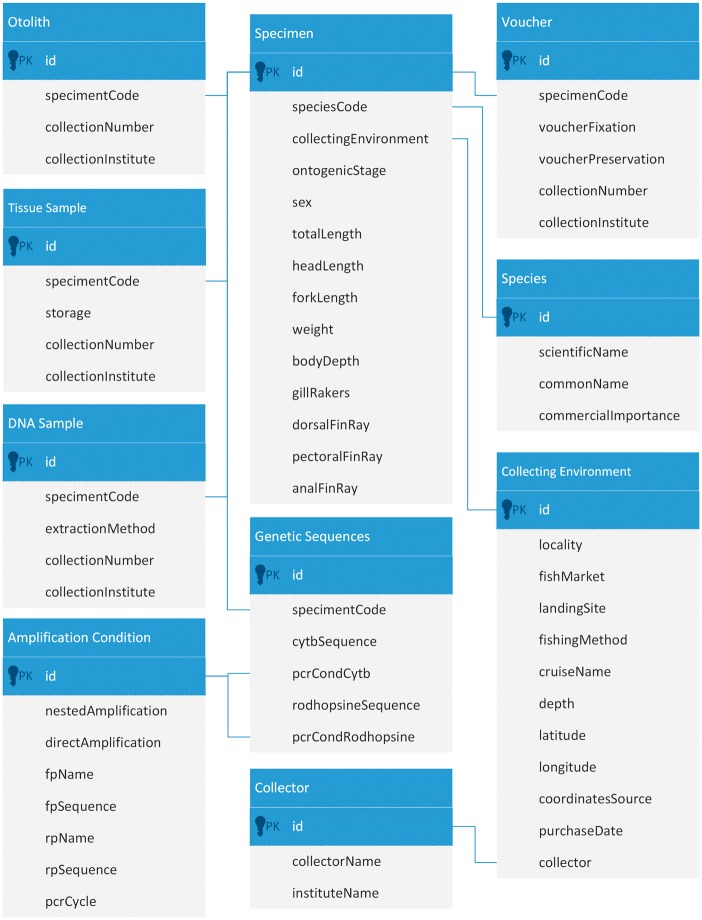
Extract from the database schema of the FishTrace catalogue showing the main tables and their relationships.

The unique code for the specimens has been used in the database design to ensure an unambiguous link between data about vouchers, tissues, otoliths and genetic sequences as can be seen in the subset of the database schema (Figure 3) for the FishTrace catalogue containing the main tables and their relationships.

The table called ‘Collecting Environment’ describes the procedure followed in the sampling of each fish specimen in the field, including the fishing method and the landing site. This table also contains the geographical coordinates in decimal degrees, which allow the visualization of the data on a geographical map, and a link to the ‘Collector’ table containing references to the persons that collected the samples.

The table called ‘Specimen’ contains all the taxonomic information obtained from each fish specimen analysed within FishTrace. It includes specimen length, weight, ontogenic stage, information on gill rakers and fin rays. Each specimen is linked to an entry in the ‘Collecting Environment’ table, and several specimens can be linked to the same environment entry.

The table ‘Voucher’ contains information on the vouchers included into the reference collections; i.e. the FishTrace code of the specimen, the fixation substance used, the preservation medium used, the voucher collection number and the collection institute. The table ‘Tissue Sample’ contains information on the tissues extracted from each fish specimen collected; i.e. the FishTrace code of the specimen, the storage medium used, the tissue collection number and the collection institute. Several tissue samples can be available for a single specimen. The table ‘Otolith’ contains information related to the otoliths extracted from each fish specimen collected; i.e. the FishTrace code of the specimen, the otolith collection number and the collection institute.

The purpose of the table ‘Amplification Condition’ is to describe the PCR conditions followed to amplify both targeted genes; whereas the table ‘Species’ lists the names of the species with information about the commercial interest of each species.

Finally, the table called ‘Genetic Sequences’ contains the genetic information obtained from each fish specimen analysed within FishTrace. It includes the complete cytochrome b and rhodopsin sequences with links to the PCR conditions used. 

## Data dissemination

Data from the FishTrace database are currently disseminated on the FishTrace web site through Tableau, a data visualization platform providing functionalities to visualize data and to perform business intelligence analysis. Data can also be exported in CSV (Comma Separated Values) format, which can be used with spreadsheet programs.

On the FishTrace web site the data visualization page is structured in three main sections, respectively, called: ‘Sampling Map’, ‘Collections’ and ‘DNA Sequences’. Moreover, in the ‘Collections’ section, information about the collections is grouped in different dashboards that visualize data on vouchers, otoliths, tissue samples and DNA samples, respectively.

As explained before, the unique FishTrace specimen code (its format has been described in the previous paragraph) has been used in the database to connect data about vouchers, tissue samples, otoliths, DNA samples and sequences; it has therefore been used also in the visualization of the data as main connection among the different sections and dashboards.

On the web site, the FishTrace data are mainly accessed through filtering functionalities, resulting in a user-friendly approach for the user that can explore the data on the basis of different criteria. Search options are also available to allow the direct access to a specific sample or collection item when the sample code or the collection number is already known by the user.

**Figure 4.**
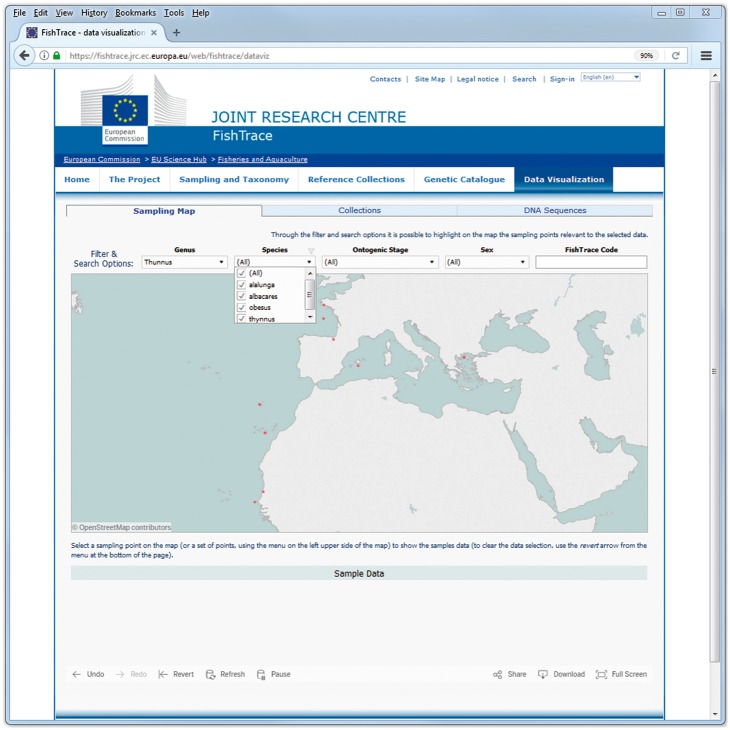
Sampling Map data visualization page–data filtering.

**Figure 5.**
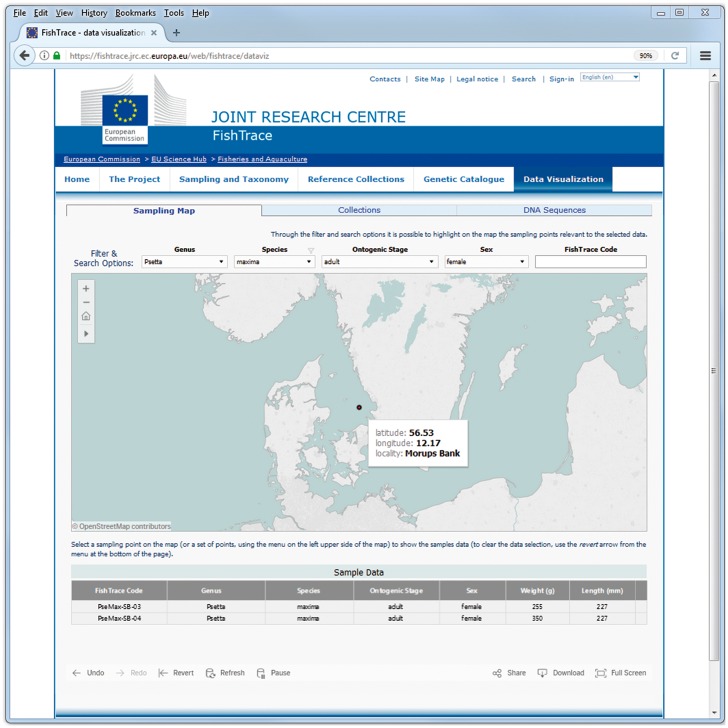
Sampling Map data visualization page–selection of a sampling point.

On the ‘Sampling Map’ page, the geographical coordinates of the samples along with some information about the samples themselves are presented. When filtering on genus, species, ontogenic stage or sex values, only the sampling points corresponding to the selected samples are shown on the map (Figure 4). Whereas when selecting a sampling point (or a set of sampling points) on the map, information about all the samples from that location (or locations) are visualized (Figure 5): FishTrace code, genus, species, ontogenic stage, sex, weight and length are shown. The FishTrace code can then be used to retrieve, from the other pages, genetic data and/or the biological materials that have been stored in the collections for the selected sample.

**Figure 6.**
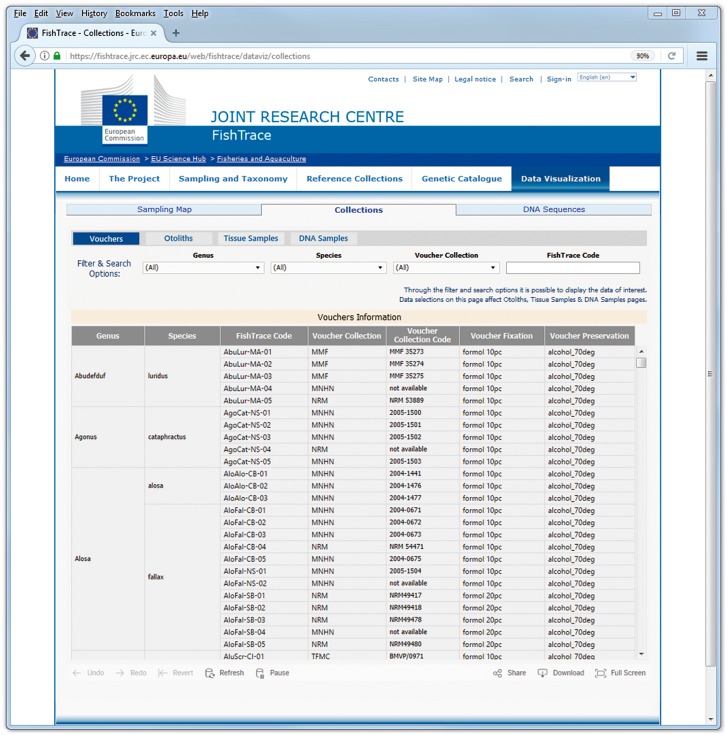
Collections data visualization page.

On the *‘*Collections’ pages (Figure 6) it is possible to filter the data on genus, species and on the museum hosting the biological collection or to search a collection item using the FishTrace code of its associated sample. On these pages it is possible to retrieve the FishTrace code that can be then used to retrieve data from other pages or to find the geographical position of the sample from the map page.

On all the ‘Collections’ pages the collection number code is also provided, which can be used to request the corresponding sample from the museum where it is preserved; this collection number is required in the loan form that has to be directed to the curator of the collection which the chosen sample belongs.

**Figure 7.**
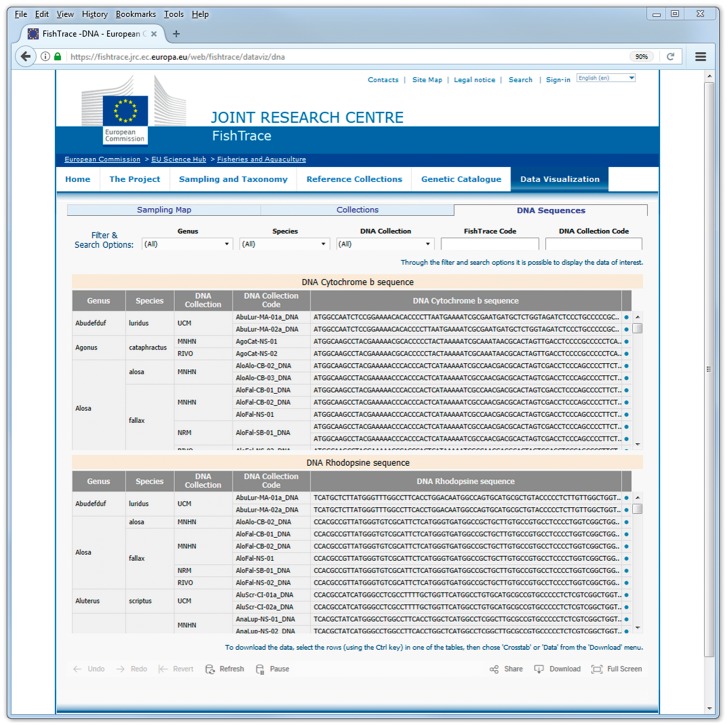
DNA sequences data visualization page.

On the *‘*DNA Sequences’ page (Figure 7) it is possible to filter the data on genus, species and on the museum hosting the biological collection. An alternative, using the search options available for the FishTrace code and for the ‘DNA Collection’ code it is possible directly access the sequences derived from a specific sample or collection item. Also on the ‘DNA Sequences’ page it is possible to retrieve the FishTrace code that can be used to link to data from other pages or to find the geographical position of the sample from the map page.

**Figure 8.**
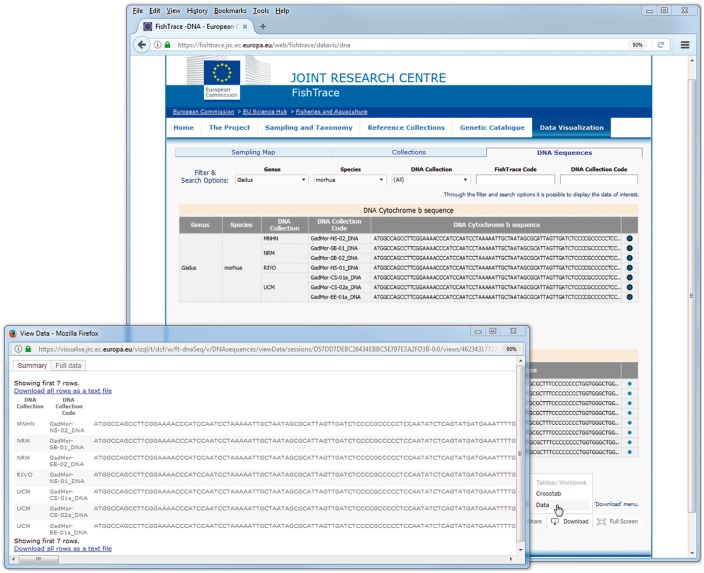
DNA sequences data visualization page–data download.

In the ‘DNA Sequences’ page, data can be downloaded clicking on the ‘Download’ button at the right bottom of the page and choosing the option ‘Data’ or *‘*Crosstab’; from the new window that is displayed by the system, data can then be exported as a text file in CSV format (Figure 8). The download functionality by default applies on all the data visualized; in order to download only a subset of the data, it is necessary to select (with the mouse and the Ctrl key) the rows of interest before clicking the ‘Download’ button.

## Related work

The application of genetic analyses for species identification in the seafood sector, but also for fisheries management purposes, has seen an increasing interest in the last decade, due to the progress in DNA sequencing and the power of genetic marker analysis.

Initiatives, such as the Barcode of Life Data system–BOLD (11), which includes the Fish Barcode of Life–Fish-BOL (12), focus on a DNA-based identification system using a relatively small sequence fragment (648 bp) from the mitochondrial cytochrome c oxidase subunit I (COI) gene. This mitochondrial gene is very effective in the identification of the vast majority of fish species (13); however, with mitochondrial sequences it is sometimes difficult to differentiate closely related species and to identify hybrid individuals (12). In these cases, the efficiency of barcoding can be improved by targeting more than one genetic positions (14). This approach was followed in FishTrace, where both a mitochondrial gene and a nuclear gene were used, and in other projects, as for example in (15) and in the AquaGene database (16); in the latter, fish species are genetically characterized by two mitochondrial genes (COI and cytochrome b) and a nuclear gene (myosin 6). As an alternative to this approach, in recent studies new nuclear barcoding targets, tailored to flat fishes, have been proposed in order to approach the identification of hybrid individuals and fish species in mixtures (17).

Different degrees of data curation characterize the genetic data repositories containing DNA sequences that are currently accessible online. Curated data sources, where all the data are associated to expert-verified and vouchered material, provide data on a relative small number of species, often focusing on a taxon, as AquaGene and FishTrace itself. On the other side, open data sources as BOLD and NCBI GenBank^®^, where everybody can submit data, provide data for a large number of species; however, part of the available sequences lack validation in the form of voucher information.

The importance of vouchering morphological specimens in natural history collections for proper barcoding has been recently highlighted (18). The advantage in storing voucher specimens in museum collections is not only the morphological validation of the species; it also enables the access to samples from other projects, and it can offer the future possibility to add other barcoding markers without the need to sample again the same species.

Increasingly, natural history museums are establishing biobanks (DNA bank and/or tissue banks) for curated long-term conservation of molecular samples; some of them are also making information on their specimens available online through biobank networks like GGBN (19)–the Global Genome Biodiversity Network–increasing the awareness of the availability of these resources.

Depending on the data use purpose, the geographical origin of the specimens from where DNA was extracted can be a relevant aspect. FishTrace interest focused on the European waters; the AquaGene collection of samples originates from the Central Eastern Atlantic off the coast of West Africa; whereas FISH-BOL and GGBN have a much wider geographical target. However, European waters and species of interest for the fisheries in Europe are not yet well represented in GGBN, where fish specimens are mostly provided by the Natural History Museum at the University of Oslo (20) and the Ocean Genome Legacy Center (21).

## Conclusion

The FishTrace genetic catalogue presented here, is a genetic database where every DNA sequence identifying a species is unambiguously linked to a voucher that is independently identified by taxonomists and curated by natural museums. This feature is an invaluable asset for species identification of seafood in a legal context and a solid basis for future research on genetic species identification of fish and fish products. That this is a pertinent issue, is shown by recent publications (e.g. 17, 22, 23), and is emphasized in the European Union, as well as international legislation (1). The successful exploitation of the FishTrace genetic catalogue as a reference database for policy-linked research (17) prompted us to publish this well-curated database as to enhance awareness and use.

The persistent availability and quality assurance of the FishTrace genetic catalogue is well demonstrated. It has been hosted and managed by the European Commission Joint Research Centre (JRC) since the end of the FishTrace project in 2006. After a revision of the quality of the data stored in the database in 2008 by the Complutense University of Madrid and a number of updates to the database structure, in 2017 the FishTrace web site was overhauled and a new software facility to access the data has been provided. FishTrace is one of very few examples of a publicly funded database underpinning fish and fishery research as well as management and conservation, that persists well beyond the lifetime of the project itself.

The FishTrace database is of high relevance not only for research but also for policy support. An example is control and enforcement in the fisheries sector. Exploitation of fish and shellfish stocks by the European Union fishing fleet is managed under the Common Fisheries Policy (CFP), which aims to ensure that fishing and aquaculture are environmentally, economically and socially sustainable and that they provide a source of healthy food for European citizens; in this context FishTrace enables and can support traceability schemes (24) as required under Article 13 of the CFP control regulation (EC) 1224/2009.

## References

[1] Hofherr J., Martinsohn J.T., Cawthorn D. (2016) Chapter 3–regulatory frameworks for seafood authenticity and traceability in seafood authenticity and traceability In: Naaum A.M., Hanner R.H. (eds). Seafood Authenticity and Traceability. Academic Press, London, pp. 47–82.

[2] http://www.pescabase.org/doc_html/frameset_pb.html (31 July 2017, date last accessed).

[3] NCBI Resource Coordinators. (2016) Database resources of the National Center for Biotechnology Information. Nucleic Acids Res., 44, D7–D19. 26615191 10.1093/nar/gkv1290 PMC4702911

[4] Nelson J.S. (2006) Fishes of the World, 4th edn. Wiley, New York, p. 601.

[5] Sevilla R.G., Diez A., Noren M. (2007) Primers and polymerase chain reaction conditions for DNA barcoding teleost fish based on the mitochondrial cytochrome b and nuclear rhodopsin genes. Mol. Ecol. Notes, 7, 730–734.

[6] Streit B., Stadler T., Schwenk K. (1994) Natural hybridization in freshwater animals. Ecological implications and molecular approaches. Naturwissenschaften, 81, 65–73. 8145857 10.1007/BF01138462

[7] Dettai A., Lecointre G. (2005) Further support for the clades obtained by multiple molecular phylogenies in the acanthomorph bush. C. R. Biol., 328, 674–689. 15992750 10.1016/j.crvi.2005.04.002

[8] Shedlock A.M., Haygood M.G., Pietsch T.W. (1997) Enhanced DNA extraction and PCR amplification of mitochondrial genes from formalin-fixed museum specimens. Biotechniques, 22, 394–396. 398, 400. 9067006 10.2144/97223bm03

[9] Chakraborty A., Sakai M., Iwatsuki Y. (2006) Museum fish specimens and molecular taxonomy: a comparative study on DNA extraction protocols and preservation techniques. J. Appl. Ichthyol., 22, 160–166.

[10] Lychakov D.V., Rebane Y.T. (2000) Otolith regularities. Hear. Res., 143, 83–102. 10771186 10.1016/s0378-5955(00)00026-5

[11] Ratnasingham S., Hebert P.D.N. (2007) BOLD: the barcode of life data system. Mol. Ecol. Notes, 7, 355–364. 18784790 10.1111/j.1471-8286.2007.01678.x PMC1890991

[12] Ward R.D., Hanner R., Hebert P.D.N. (2009) The campaign to DNA barcode all fishes, FISH-BOL. J. Fish Biol., 74, 329–356. 20735564 10.1111/j.1095-8649.2008.02080.x

[13] Ward R.D. (2009) DNA barcode divergence among species and genera of birds and fishes. Mol. Ecol. Resour., 9, 1077–1085. 21564845 10.1111/j.1755-0998.2009.02541.x

[14] Ardura A., Planes S., Garcia-Vazquez E. (2013) Applications of DNA barcoding to fish landings: authentication and diversity assessment. ZooKeys, 365, 49–65. 10.3897/zookeys.365.6409 PMC3890670 24453550

[15] Hanner R., Floyd R., Bernard A. (2011) DNA barcoding of billfishes. Mitochondrial DNA, 22 Suppl 1, 27–36. 10.3109/19401736.2011.596833 21980985

[16] http://www.aquagene.org (31 July 2017, date last accessed).

[17] Paracchini V., Petrillo M., Lievens A. (2017) Novel nuclear barcode regions for the identification of flatfish species. Food Control, 79, 297–308. 28867876 10.1016/j.foodcont.2017.04.009 PMC5446357

[18] Astrin J.J., Zhou X., Misof B. (2013) The importance of biobanking in molecular taxonomy, with proposed definitions for vouchers in a molecular context. In: Nagy,Z.T., Backeljau,T., De Meyer,M. and Jordaens,K. (eds). DNA barcoding: a practical tool for fundamental and applied biodiversity research. ZooKeys, 365, 67–70. 10.3897/zookeys.365.5875 PMC3890671 24453551

[19] Droege G., Barker K., Astrin J.J. (2013) The global genome biodiversity network (GGBN) data portal. Nucleic Acids Res., 42, D607–D612. 24137012 10.1093/nar/gkt928 PMC3965106

[20] http://www.nhm.uio.no (31 July 2017, date last accessed).

[21] http://www.northeastern.edu/ogl (31 July 2017, date last accessed).

[22] Miller D., Jessel A., Mariani S. (2012) Seafood mislabelling: comparisons of two western European case studies assist in defining influencing factors, mechanisms and motives. Fish Fisheries, 13, 345–358.

[23] Helyar S.J., Lloyd H.A.D., De Bruyn M. (2014) Fish product mislabelling: failings of traceability in the production chain and implications for Illegal, Unreported and Unregulated (IUU) fishing. PLoS ONE, 9, e98691. 24921655 10.1371/journal.pone.0098691 PMC4055496

[24] Martinsohn J.T. (2011) Deterring Illegal Activities in the Fisheries Sector–Genetics, Genomics, Chemistry and Forensics to Fight IUU Fishing and in Support of Fish Product Traceability. JRC Reference Reports. European Commission. Luxemburg. ISBN 978-92-79-15905-3.

